# 

**Published:** 1899-12-30

**Authors:** 


### NOTICE TO CORRESPONDENTS.

**All MS., Letters, Books for Review, and other matters intended for the Editor should be addressed The Editor, "The Hospital," The Hospital Building, 28 & 29, Southampton Street, Strand, W.C.**

**The Editor cannot undertake to return rejected MS., even when accompanied by stamped directed envelope.**

**Notice to Advertisers and Subscribers.**

**All Advertisements, Orders for Copies of The Hospital, and Business Communications should be addressed to THE MANAGER (Not to the Editor), The Hospital, The Hospital Building, 28 & 29, Southampton Street, Strand, London, W.C.**

**The Cost of Subscription, post free, is as follows:—Per week, 2½d.; per quarter, 2s. 8d.; per half-year, 5s. 3d.; per annum, 10s. 6d. Foreign:—Per annum, 15s. 2d., payable, in all cases, in advance.**

**NOTICE.—Vol. XXVI. of "The Hospital" (April, 1899, to September, 1899), in handsome cloth binding, is now on sale at the Office of this Journal, price 7s. 6d. Cases for binding the Numbers contained in this Volume 1s. 6d. Index to Vol. XXVI., 2½d., post free.**

**The Monthly Part for January will be ready on 1st January, 1900. Price 6d., by post 8d.**

### BOOKS RECEIVED.

William F. Clay.

"Lectures on Hœmorrhage and Eclampsia." By Robert Jardine, M.D.
"Clinical Studies in Vice and Insanity." By George R. Wilson, M.D.

Adam and Charles Black.

"Who's Who," 1900.
"The Englishwoman's Year Book," 1900. Edited by Emily Janes.

E. Merck, Darmstadt.

"Merck's Manual of Materia Medica."

It is comforting news to learn through the special correspondent of the *British Medical Journal* that the organisation of the Army Medical Corps for the sick and wounded is working excellently. From the collecting stations to the base hospitals everything is reported as working smoothly and well.

### THE STUDENT OF MEDICINE.

**APPOINTMENTS.**

J. Collier, M.D., M.R.C.P., B Sc., appointed Medical Registrar to the National House, Queen Square, W.C. N. Colville, M.B.Toronto, appointed Clinical Assistant to the Chelsea Hospital for Women. L Gibbs, M.R.C.S., L.R.C.P., appointed Clinical Assistant to the Hospital for Diseases of the Skin, Blackfriars. G. C. Hayes, F.R.C.S.Eng., appointed Assistant Ophthalmic and Aural Surgeon to the Leeds General Infirmary. A. A. Hill, L.R.C.P., L.R C.S.Edin., appointed Certifying Factory Surgeon for Tunstall and District. T. H. Littlejohn, M.B., C.M., D P.H.Edin., appointed Medical Officer of Health to the Redditch Urban District Council. A. F. MacCallan, M.B., B.C.Cantab., F.R.C.S Eng, appointed Junior House Surgeon at the Royal London Ophthalmic Hospital. R. J. Owen, L.R.C.P.Lond., M.R.C.S.Eng., appointed Medical Officer of Health for the Llandovery Town Council. W. B. Parsons, M R C.S., L.R.C.P.Lond., appointed Resident Medical Officer to the North West London Hospital, Kentish Town Road C. H. Russell, M.R.C.S., L.R.C P., D.P.H.Lond., appointed Medical Officer of Health for Great Yarmouth. S. V. H. Underhill, L.R.C P., M.R.C.S., appointed House Surgeon to the Suffolk General Hospital.

Queen Charlotte's Hospital, Marylebone, N.W.—The following appointments have been made in the Medical Staff: Consulting Physician: Dr. W. C. Grigg. Physicians to In-patients: Dr. W. R. Pollock and Dr. W. J. Gow. Physician (Non-Obstetric): Dr. W. P. Herringham. Physicians to Out Patients: T. W. Eden, M D.Edin, M.R.C P.Lond.; C. H. Roberts, M.D., M.R.C.P.Lond.; A. F. Stabb, M.B., B.C.Camb, M.R.C.P.Lond, M.R.C.S.Eng. Ophthalmic Surgeon: S. Stephenson, M.B., C.M.Edin., F.R C.S.Edin. Dental Surgeon: F. Morley, M.R.C.S.Eng., L.R.C.P.Lond, L.D.S.Eng.

Royal 8vo., illustrated by a series of Original Photo-micrographs and many Illustrations in the Text, cloth gilt, 7s 6d. net,

THE MENOPAUSE AND ITS DISORDERS.

With Chapters on Menstruation.

By A. D. LEITH NAPIER, M.D., M.R.C.P., late Editor of the *British Gynæcological Journal.*

London: The Scientific Press, Ltd., 28 & 29, Southampton Street, Strand, London, W.C.

NOW READY. With 36 Illustrations, crown 8vo., cloth, 248 pp., price 6s.

### PRACTICAL TEXT-BOOK OF MIDWIFERY FOR NURSES AND STUDENTS.

By ROBERT JARDINE, M.D.Ed., M.R.C.S.Eng,, F.F.P. & S.Glasgow, Physician to the Glasgow Maternity Hospital.
By the Same Author.

**CLINICAL LECTURES ON THE HÆMORRHAGES OF PREGNANCY, PARTURITION, AND THE PUERPERIUM,**
AND THE TREATMENT OF ECLAMPSIA BY SALINE INFUSION, with Notes on 17 Cases.
84pp. 8vo., price 1s. 6d., by post 1s. 8d.

WILLIAM F. CLAY, 18, TEVIOT PLACE, EDINBURGH.

Demy 8vo., with two Plates, cloth gilt, 12s. 6d. net.

**MEDICAL HISTORY FROM THE EARLIEST TIMES.**

By E. T. WITHINGTON, M.A., M.B.Oxon.

"One of the best attempts that has yet been made in the English language to present the reader with a concise epitome of the history of medicine, and as such we very cordially commend it."—*Glasgow Medical Journal.*

London: THE SCIENTIFIC PRESS, Ltd., 28 & 29, SOUTHAMPTON STREET, STRAND, W.C.

### NOW READY,

A REPRINT OF

### A NURSING HANDBOOK

TOGETHER WITH S. MAW, SON, AND THOMPSON'S

### COMPLETE CATALOGUE OF NURSING REQUISITES.

The above work is now republished at a cost of 1s. per copy. Messrs. S. M., S., and T. have been compelled to make this charge on account of the **extreme popularity** of the work and the great expense incurred in its production.

***Post Free on receipt of 1s.***

### S. MAW, SON, and THOMPSON,

7 to 12, ALDERSGATE STREET, LONDON, E.C.

